# Computational KIR copy number discovery reveals interaction between inhibitory receptor burden and survival

**Published:** 2019

**Authors:** Rachel M. Pyke, Raphael Genolet, Alexandre Harari, George Coukos, David Gfeller, Hannah Carter

**Affiliations:** School of Medicine, University of California, San Diego, 9500 Gilman Dr. San Diego, CA 92093, USA, ramarty@ucsd.edu; Ludwig Institute for Cancer Research, University of Lausanne, Chemin des Boveresses 155 Epalinges, VD, CH, 1066; Ludwig Institute for Cancer Research, University of Lausanne, Chemin des Boveresses 155 Epalinges, VD, CH, 1066; Ludwig Institute for Cancer Research, University of Lausanne, Chemin des Boveresses 155 Epalinges, VD, CH, 1066; Ludwig Institute for Cancer Research, University of Lausanne, Chemin des Boveresses 155 Epalinges, VD, CH, 1066; School of Medicine, University of California, San Diego, 9500 Gilman Dr. San Diego, CA 92093, USA hkcarter@ucsd.edu

**Keywords:** Killer immunoglobulin-like receptors, KIR, cancer, immunology, MHC, copy number

## Abstract

Natural killer (NK) cells have increasingly become a target of interest for immunotherapies1. NK cells express killer immunoglobulin-like receptors (KIRs), which play a vital role in immune response to tumors by detecting cellular abnormalities. The genomic region encoding the 16 KIR genes displays high polymorphic variability in human populations, making it difficult to resolve individual genotypes based on next generation sequencing data. As a result, the impact of polymorphic KIR variation on cancer phenotypes has been understudied. Currently, labor-intensive, experimental techniques are used to determine an individual’s KIR gene copy number profile. Here, we develop an algorithm to determine the germline copy number of KIR genes from whole exome sequencing data and apply it to a cohort of nearly 5000 cancer patients. We use a k-mer based approach to capture sequences unique to specific genes, count their occurrences in the set of reads derived from an individual and compare the individual’s k-mer distribution to that of the population. Copy number results demonstrate high concordance with population copy number expectations. Our method reveals that the burden of inhibitory KIR genes is associated with survival in two tumor types, highlighting the potential importance of KIR variation in understanding tumor development and response to immunotherapy.

## 1. Introduction

Killer Immunoglobulin-like receptors (KIRs) are cell-surface receptors expressed by Natural Killer (NK) cells and some T cells. KIRs bind to other naturally occurring immune receptors, including Major Histocompatibility Complexes (MHCs), to inhibit or activate immune cell activity^2^. MHC molecules, which are expressed on nearly all nucleated cells, can present pathogenic or tumorigenic peptides on the cell surface for recognition by T cells. In order to evade the immune system, malignant cells often down regulate expression of MHC molecules^3^. However, KIR on NK cells are able to respond with an immune attack if they can recognize that the expression of MHC deviates from normal^4^. This dual system allows “no way out” for cancerous cells -- either the MHC presents the neo-peptides or the MHC is downregulated and NK cells attack the cell^5^. However, the efficiency of this process depends greatly on the ability of the KIR expressed on NK cells to bind to the MHC receptors.

The impact of these NK cell mechanisms in response to malignancies has been validated through the several associations found between KIR genotype and cancer phenotypes. The presence of certain KIR genes can predict response to immunotherapy treatment and survival outcomes in chronic myeloid leukemia and acute myeloid leukemia^6,7^. Associations have also been found between specific KIR genes and susceptibility to several cancers (malignant melanoma, leukemia, nasopharyngeal carcinoma, and cervical cancer)^5,8–10,11^. Furthermore, the strength of HLA-KIR interactions plays a functional role and can influence disease susceptibility^12^.

However, all of these studies have been performed on cohorts of low sample size due to the difficulty of studying the highly variable KIR region. KIRs are encoded by a cluster of genes on chromosome 19q13.4. Individuals vary widely in the number of KIR genes they carry and in the allelic variation within those genes. The region can contain up to 16 genes but sometimes has as few as four gene, each one with up to 100 known allelic variants.

The highly homologous nature of the KIR genes hampers usage of conventional, computational copy number technologies for short read Next Generation Sequencing (NGS) data. However, the interesting immune implications of the region have led to the development of several experimentally based techniques. One approach uses polymerase chain reaction to amplify the sequences and sequence specific primers to detect particular alleles^13^. Another uses sequence specific oligonucleotides as a first pass and then sequences specific exons to identify allelic variation^14^. Sanger sequencing can also provide long enough reads to cover several genes at a high resolution^14,15^. However, all of these techniques require KIR specific techniques in the data gathering stage. Only two computational alternatives exist that do not require KIR specific techniques in the data gathering stage. KIR*IMP imputes the KIR region from SNP genotype data^16^ and PING predicts KIR copy number from NGS data^17^. However, KIR*IMP cannot be applied to large exome datasets and PING requires time consuming read mapping, a potentially biased normalization and manual curation step.

**Figure 1.**
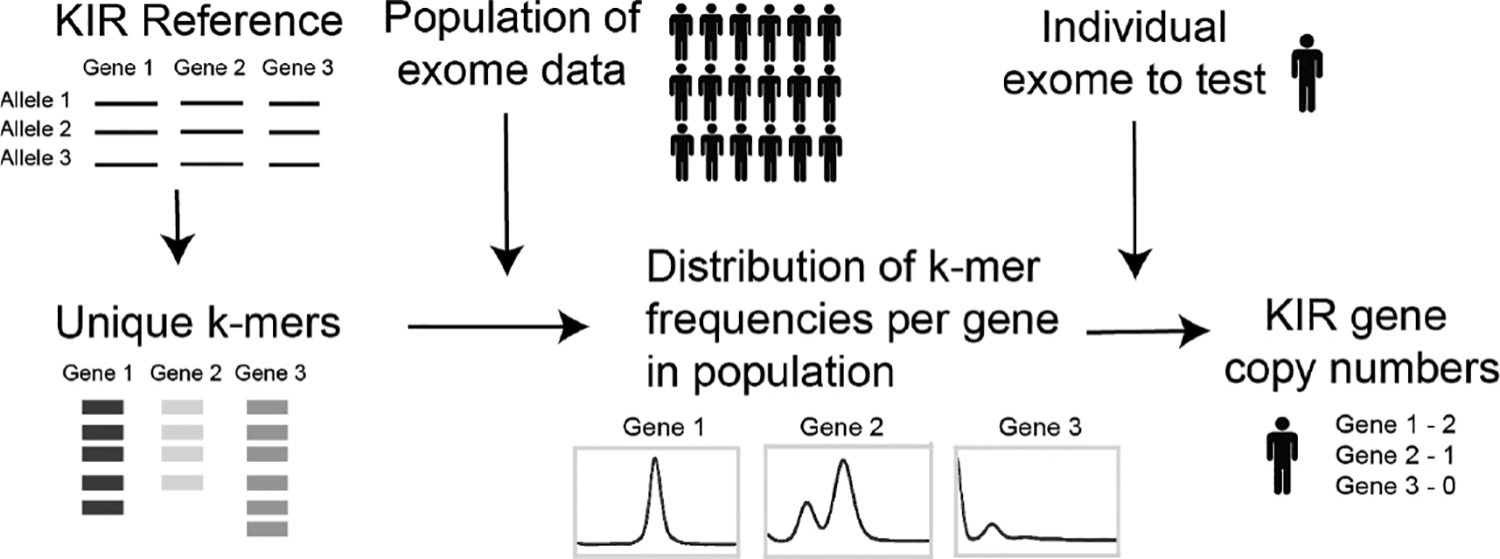
Schematic of copy number calling pipeline. Unique k-mers are derived from a KIR reference library. The exome data for thousands of individuals is searched for these unique k-mers to find distributions of frequencies in the population. The copy number for a specific individual can be deduced from where their frequency falls in the distribution.

To achieve the computational speed and accuracy required for inferring the KIR types of nearly six thousand cancer patients in order to study tumor phenotypes, we implemented an unsupervised, k-mer based algorithm that leverages large populations to determine copy number (Figure 1). Using this cancer cohort, we discovered that patients in uterine and cervical cancer survive longer when they have fewer inhibitory KIR genes as compared to patients that have more inhibitory genes.

## 2. Materials and Methods

### 2.1 Data collection

Exome sequencing, transcriptome sequencing and clinical data from The Cancer Genome Atlas was downloaded from the National Cancer Institute’s Genomic Data Commons on August 3rd, 2018. All disease types were obtained. KIR alleles were downloaded from the Immuno Polymorphism Database on October 6th, 2016^18^. Population KIR allele frequencies were obtained from The Allele Frequency Net Database on February 22, 2017^19^.

### 2.2 K-mer selection

**Figure 2.**
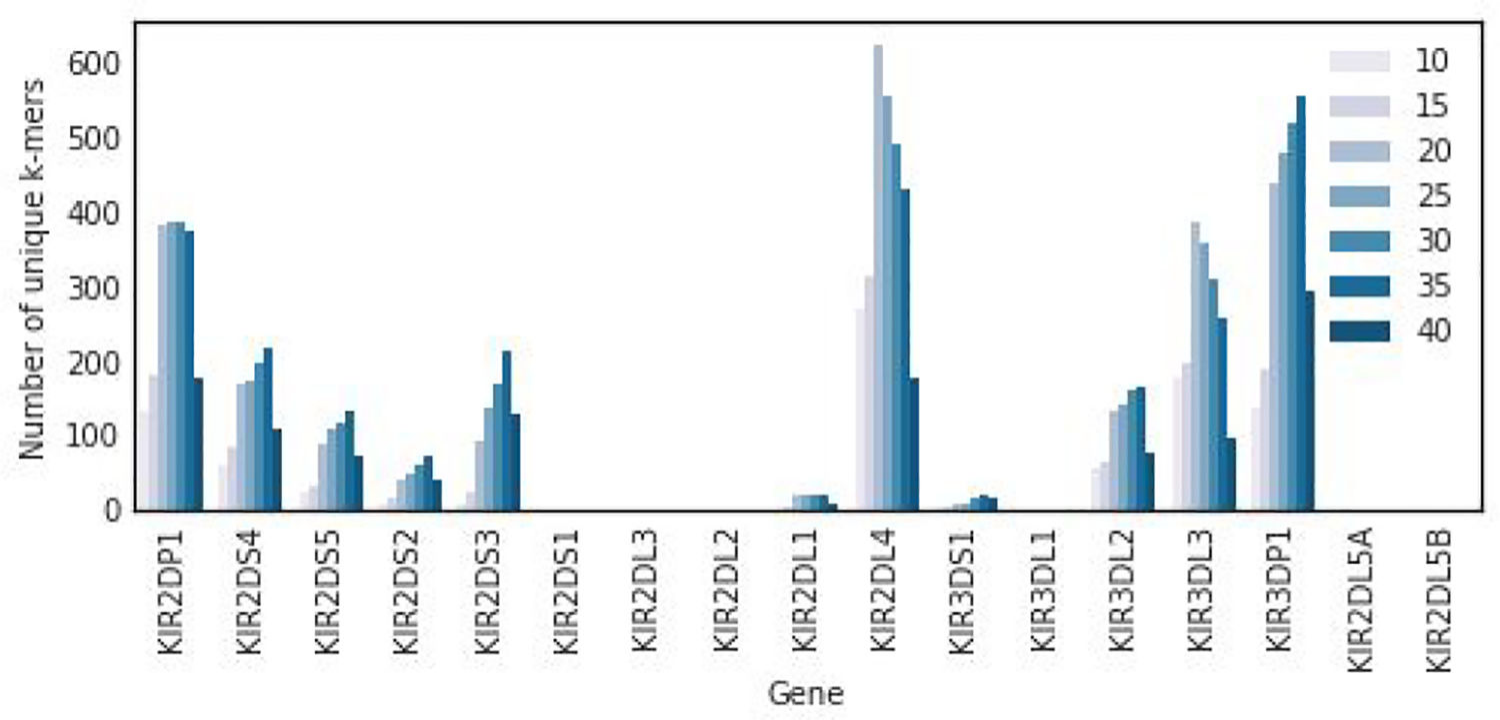
Unique k-mer counts. The number of unique k-mers found in each KIR gene across a spectrum of k.

A set of k-mers were selected to represent each KIR gene -- these k-mers are referred to as unique k-mers. The criteria for the unique k-mers are as follows: a unique k-mer, or its reverse complement, must appear in (1) every allele of a specific KIR gene and (2) no alleles of any other KIR gene. Unique k-mers of lengths 10, 15, 20, 25, 30, 35 and 40 were collected based on the KIR reference from the Immuno Polymorphism Database (IPD)^18^. The number of unique k-mers for each gene is shown in Figure 2. In addition, only one length of k-mer, 30, was collected in 100 random genes from throughout the genome.

### 2.3 NGS pipeline and k-mer extraction

The genomic region encoding the KIR locus (GRCh38:chr19:54025634–55084318) and the regions encoding the 100 random genes were extracted from the exome sequencing bam files from the TCGA. The unmapped reads of the exome sequencing bam files were also pulled from the exome sequencing bam files. All of these genomic regions were merged together into a single bam file. Then, the reads were stripped into a fastq file and realigned using Bowtie2^20^ to a reference that is constructed of all the KIR alleles for each KIR gene from IPD and each of the 100 random reference genes. All reads that mapped in the reference at least once are again stripped and then searched for the set of unique k-mers and occurrence counts are stored for each k-mer. The pipeline concludes with each patient having a vector of occurrence counts for every unique k-mer.

### 2.4 Data cleaning

To identify substructure in the dataset that might indicate problematic samples, the k-mer frequency for each of a set of 100 random genes for all patients in TCGA are visualised with a t-SNE plot^21^. To further understand the relationship between sequencing depth and clusters of samples, we plotted the distribution of k-mer counts in the set of 100 random genes and also k-mer counts in the KIR region. To reduce noise from outliers, only the samples from the largest cluster of the t-SNE (Agilent Sureselect capture kit) were selected and all samples with < 40,000 k-mer coverage in the set of 100 random genes and < 20,000 k-mer coverage in the set of KIR genes were excluded. After applying these filters, a total of 4,717 samples remained.

### 2.5 Normalization of k-mer frequencies

Since every sample will have different sequencing depth, the k-mer counts must be normalized before being compared between samples. Furthermore, there are several lengths of k to choose between. We evaluated normalization methods and lengths of k based on reduction in variance of k-mer counts associated with KIR3DL3 which is known to be almost universally diploid. We tested each length of k (15, 20, 25, 30, 35, 40) against each of the following normalization approaches: (1) the mean of the number of k-mers mapped to the set of 100 random genes, (2) the mean of the number of reads with at least one k-mer mapping to the set of 100 random genes, (3) the median of the number of k-mers mapped to the set of 100 random genes and (4) the median of the number of reads with at least one k-mer mapping to the set of 100 random genes. In the end, we used a k of 30 and normalized with option (1) for the remainder of the analysis.

### 2.6 Copy number segregation and cutoff selection

**Figure 3.**
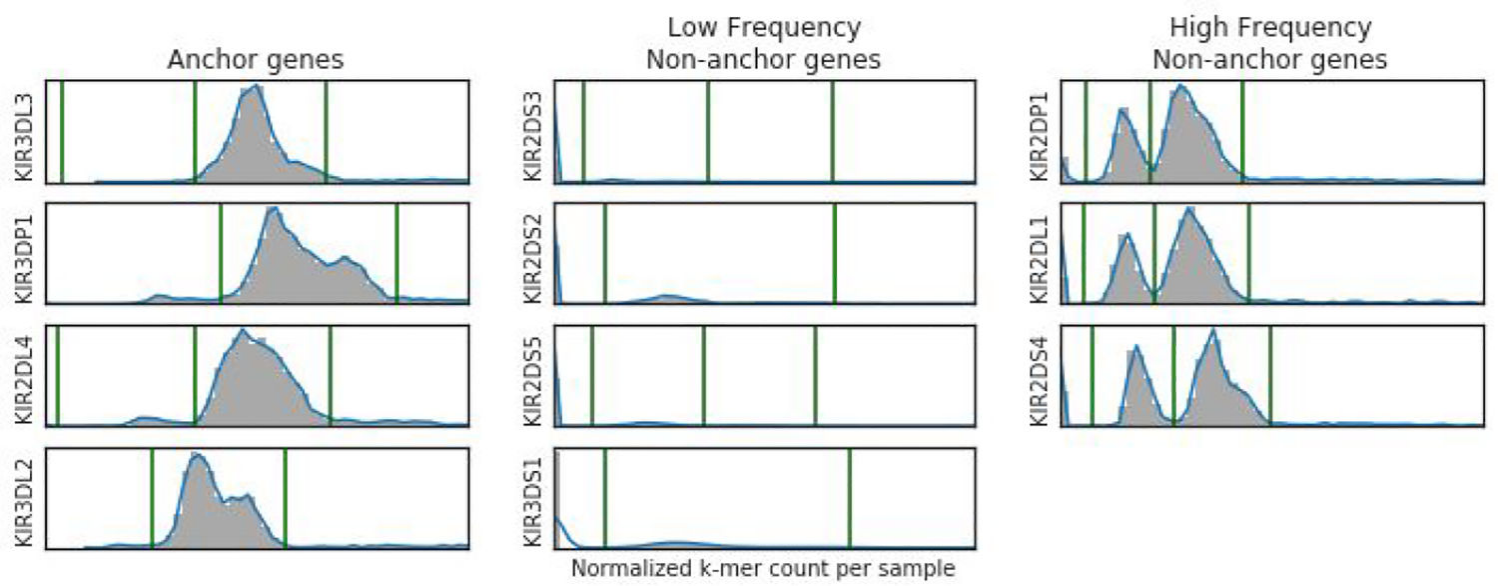
K-mer frequency distribution and copy number thresholds. The distribution of k-mer frequencies across patients in TCGA for anchor genes, high frequency non-anchor genes and low frequency non-anchor genes. The green lines denote copy number thresholds.

KIR genes have varying numbers of unique k-mers (Figure 1). After collecting 30-mer occurrences for each gene and normalizing them to the mean of the number of k-mers mapped to the set of KIR genes, we plotted the values for all individuals across the population with a histogram. Kernel density plots show the distribution of unique k-mer counts for each gene (Figure 3).

These kernel density plots can be used to assign gene copy numbers in an unsupervised manner. First, the genes are divided into three categories based on the documented ploidy of the gene: anchor genes that are present in two copies for most individuals (KIR3DL3, KIR3DP1, KIR2DL2 and KIR3DL2), high frequency non-anchor genes that are present at least once in most individuals (KIR2DP1, KIR2DL1, KIR2DS4 and KIR2DL5) and low frequency non-anchor genes that are present less than once in most individuals (KIR2DS3, KIR2DS2, KIR2DS5 and KIR3DS1). Second, peaks and valleys are called for each kernel density plot by finding local minima of the second derivative. Third, we map the highest peak to the most common ploidy based on the documented copy number variant frequency in the population and determine cutoffs by selecting the valleys surrounding that peak. For anchor genes, the highest peak is determined to be two copy numbers. Samples beyond either edge of the peak (as determined by a second derivative close to 0) are assigned a copy number of 1 or 3+. Instead of looking for subsequent minima, we used the width of the highest peak to create a new threshold for samples with 0 copies to the left of the region for 1 copy. For high frequency non-anchor genes, three peaks are usually observed and thresholds are defined as the valleys between them. The left-most and shortest peak corresponds to 0 copies, the middle peak to 1 copy and the right-most and highest peak to 2 copies. All samples beyond the the third peak correspond to 3+ copies. For low frequency non-anchor genes, typically only two peaks are observed. The left-most and highest peak is assigned 0 copies and the second peak is assigned 1 copy. The distance between these peaks is used to denote thresholds for the samples that had 2 copies or 3+ copies. Each sample was assigned copy numbers at each KIR gene according to where their k-mer count fell in the distribution. However, patients that fell very close to the cutoff boundaries for a gene (the value of the boundary that splits one copy from two copies divided by 50) were excluded for that gene. All of the genes that do not have any unique k-mers are known to co-segregate with other KIR genes. Thus, we inferred copy number for these genes from the copy number of the co-segregating gene as follows: individuals typically have as many copies of KIR2DS1 as they do KIR3DS1, KIR2DL2 as KIR2DS2, KIR3DL1 as KIR2DS4 and KIR2DL5A as the combined total of KIR2DS3 and KIR2DS5. Furthermore, individuals typically have an inverse number of KIR2DL3 as KIR2DS2 (e.g. 0 KIR2DL3 and 2 KIR2DS2, 1 KIR2DL3 and 1 KIR2DS2 or 2 KIR2DL3 and 0 KIR2DS2).

### 2.7 Validation of copy number

KIR gene counts for TCGA patients of a specific ancestry are expected to follow the documented distribution of the corresponding population. To validate this assertion, KIR gene frequencies for a European ancestry population from IPD were compared to predicted KIR gene frequencies for the European ancestry patients in TCGA. The correlation between individual gene frequencies was determined using a Pearson correlation.

### 2.8 Survival analysis

For each tumor type, we divided patients into two sets: those that had the median number of inhibitory genes or fewer and those who had greater than the median number of inhibitory genes. We calculated the survival difference between the two cohorts using the Kaplan Meier and the log rank test as implemented by the lifelines python library. P-values were adjusted with Bonferroni correction. The two tumor types with different survival outcomes, cervical squamous cell carcinoma (CESC) and uterine carcinosarcoma (UCS), were combined because of their similar physical location, immune infiltration profiles and rates in order to increase statistical power.

### 2.9 Additional immune analysis

We used RNA-seq data from TCGA to obtain immune infiltration predictions with EPIC^22^. Then, we checked the relationship between inhibitor gene count with infiltration of CD8^+^ T cells and NK cells for the tumor types where significant survival differences were found. P-values were calculated with a Mann-whitney U test between the patient set with high and low inhibitory gene counts. Furthermore, we calculated MHC-I PHBR scores (which represent the ability of a patient to present a specific mutation to the immune system based on their specific HLA alleles) for each patient’s observed driver mutations as outlined in Marty et al.^23^ and compared the PHBR scores for CESC and UCS patients with all other patients using a Mann-whitney U test.

## 3. Results and Discussions

### 3.1 Establishing unique k-mers

The key challenge for determining KIR gene copy number is the high frequency of reads mapping to multiple places across the homologous region. To address this challenge, we developed an algorithm that capitalizes on distinct k-mers to successfully determine the sequencing coverage of the gene from which each k-mer was derived. To construct our algorithm, we began by building a library of unique k-mers for all KIR genes. A unique k-mer is then defined as a string of length k that appears in *all* alleles of a specific gene but in no alleles of any other gene. The IPD contains all observed alleles of each KIR gene. Using this reference, we searched each gene for unique k-mers and found that all KIR genes either have unique k-mers (Figure 2) or are co-inherited with other KIR genes that have unique k-mers^19^.

### 3.2 Varying coverage of KIR region by exome capture kit

**Figure 4.**
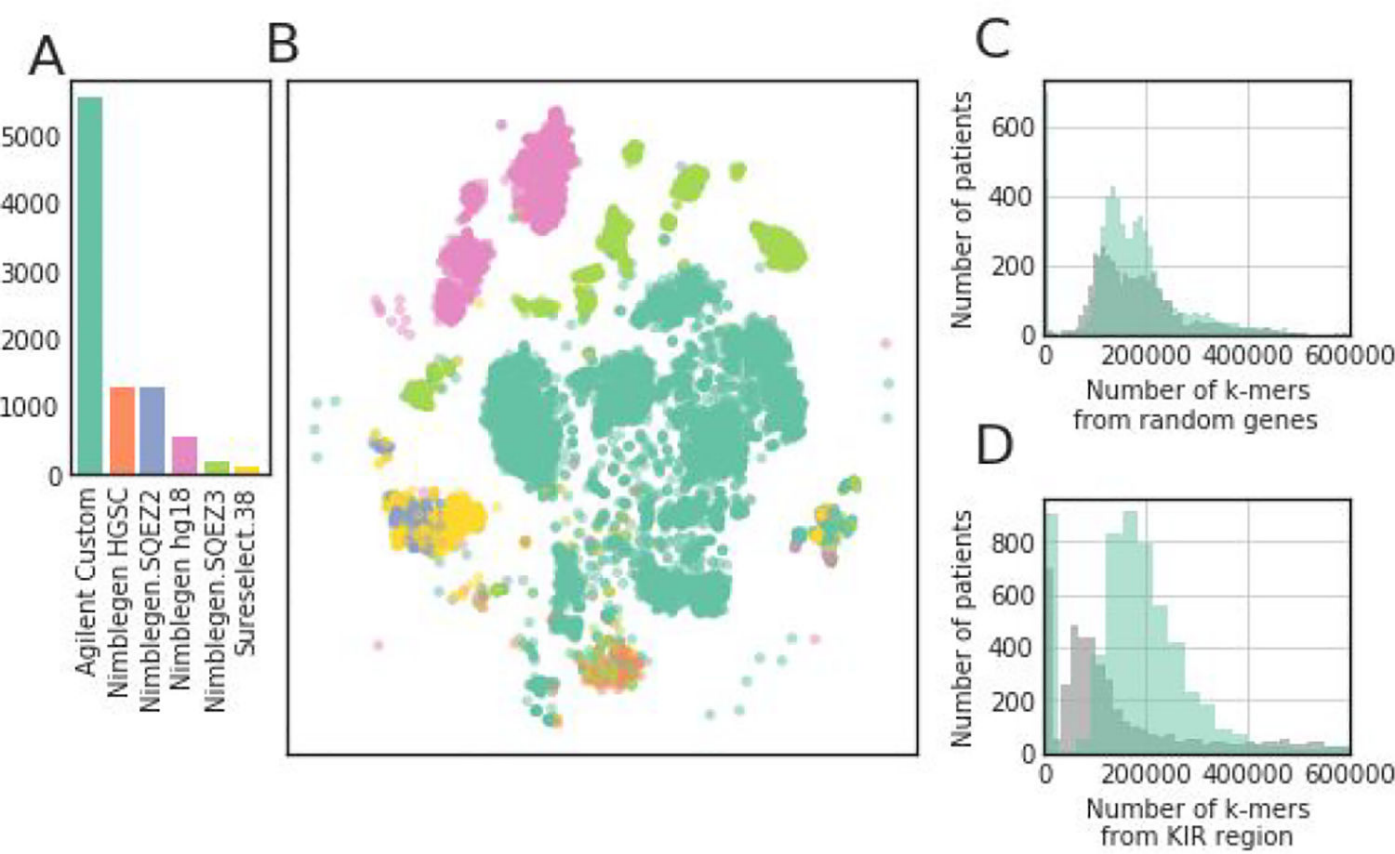
Patient exome data substructure. (A) A bar plot representing the number of patients whose exome data was captured with each exome capture kit. (B) A t-SNE plot representing the clustering of patients based on their k-mer frequency for 100 random genes in the genome. Each sample is colored by their exome capture kit. (C-D) Histograms showing the sequencing coverage of the patients with an Agilent capture kit versus the sequencing coverage of all other patients for (C) 100 random genes in the genome and (D) the KIR genes.

Next, we explored The Cancer Genome Atlas (TCGA), a large set of cancer patients (~10,000 individuals) with germline exome sequencing to learn the relationship between k-mer counts and gene copy number. We first evaluated the implication of technical covariates for our analysis. The majority of patients in TCGA had their exome captured with an Agilent capture kit; however, there were several other capture kits used for subsets of patients (Figure 4A). We selected 100 random genes in the genome and chose up to 100 unique k-mers from each gene. For each individual, we counted all observations of each k-mer and then normalized each k-mer count by the total number of observed k-mers across all 100 random genes found in that individual, resulting in a frequency for each k-mer. Using a t-SNE clustering approach, we discovered that the patients clustered by exome capture kit (Figure 4B), suggesting that capture kit could confound k-mer frequency analysis. Among capture kits, the Agilent kit was both the most frequently used kit in TCGA and the kit with the highest coverage of the KIR region. Thus we restricted our analysis to individuals sequenced with this capture kit. Furthermore, we eliminated all patients with low coverage of the 100 random genes or of the KIR region, leaving us with 4,717 high quality individuals.

### 3.3 Inference of KIR copy number

**Figure 5.**
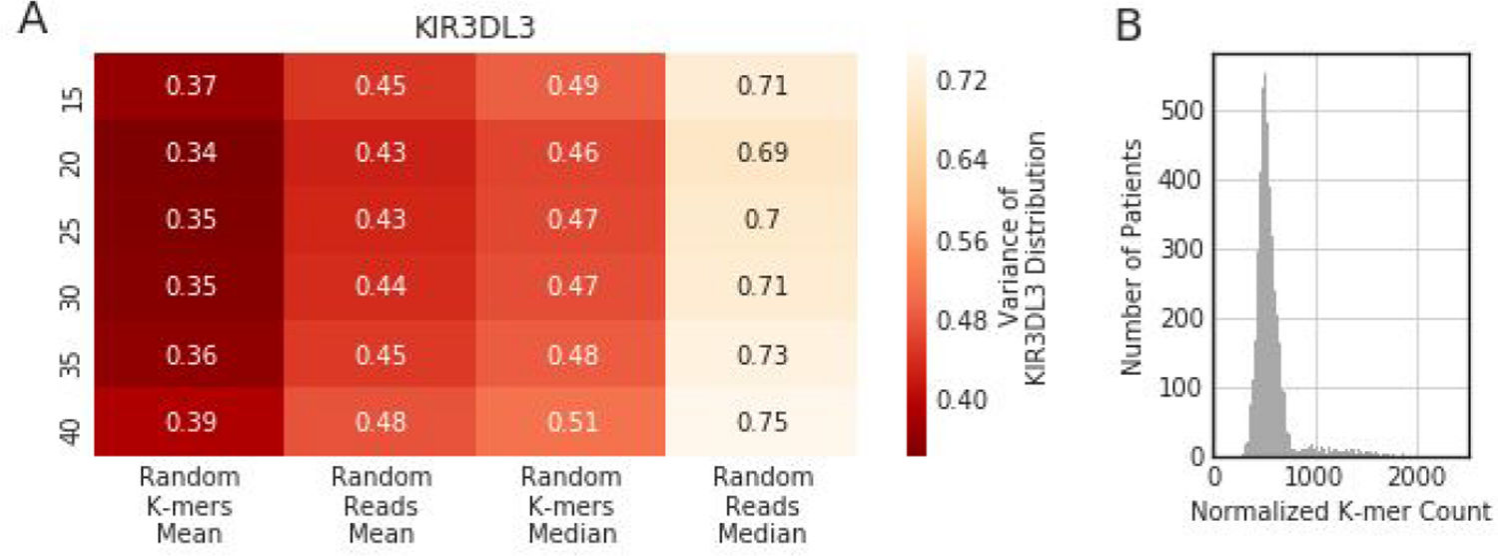
Evaluation of optimal normalization. (A) A heatmap representing the variance of k-mer frequency of KIR3DL3 anchor gene across Agilent captured TCGA patients. Several lengths of k and normalization techniques are tested. (B) A histogram showing the k-mer frequency of KIR3DL3 anchor gene with the optimal normalization technique.

Next, we searched the reads for each patient mapping to the KIR reference for unique k-mers. Since every patient will have a different sequencing depth, we had to normalize the k-mer counts before comparing them among individuals. Furthermore, we gathered k-mer counts for several lengths of k and wanted to choose the optimal value. Thus, we swept the parameter space, evaluating several normalization techniques and several values for k (Figure 5A). We evaluated each approach by determining the variance of frequency for k-mers specific to KIR3DL3 (Figure 5B), an anchor gene that is known to be present at two copies in nearly all individuals, under the assumption that lower variance across the population would mean better normalization for sequencing depth differences. We found the optimal normalization technique to be the average k-mer count of the k-mers from the 100 random genes. Though a k of 20 performed the best, we chose to k to be 30 because its performance was very close to optimal and it has higher k-mer coverage of low frequency KIR genes than a k of 20.

After establishing the normalization technique, we calculated the normalized k-mer count over all of the unique k-mers for every KIR gene of each patient. The frequencies were combined across the population to construct density curves showing the proportion of individuals with similar frequencies. Each KIR gene shows a smooth density curve with peaks that correspond to gene copy number. Anchor genes that are present in all patients have a single peak while the non-anchor genes that are present mostly at 0, 1 or 2 copies have three peaks (Figure 3). From the peaks, we determine a cutoff based around the local minima of the population densities. To determine the copy number of a specific individual, we follow the same alignment and k-mer searching approach, followed by the assignment of gene copy number depending on the individual’s placement on the curve of each gene. We applied our algorithm to 4,717 individuals in TCGA to assess the copy number of each KIR gene. For most genes, we observed good agreement to copy number calls with PING; however, on genes where the methods disagreed, our method predicted closer to the expected caucasian frequency (Figure S1A). Furthermore, our method ran four times as fast as PING on the same hardware (Figure S1B).

### 3.4 Population variation of the KIR region

**Figure 6.**
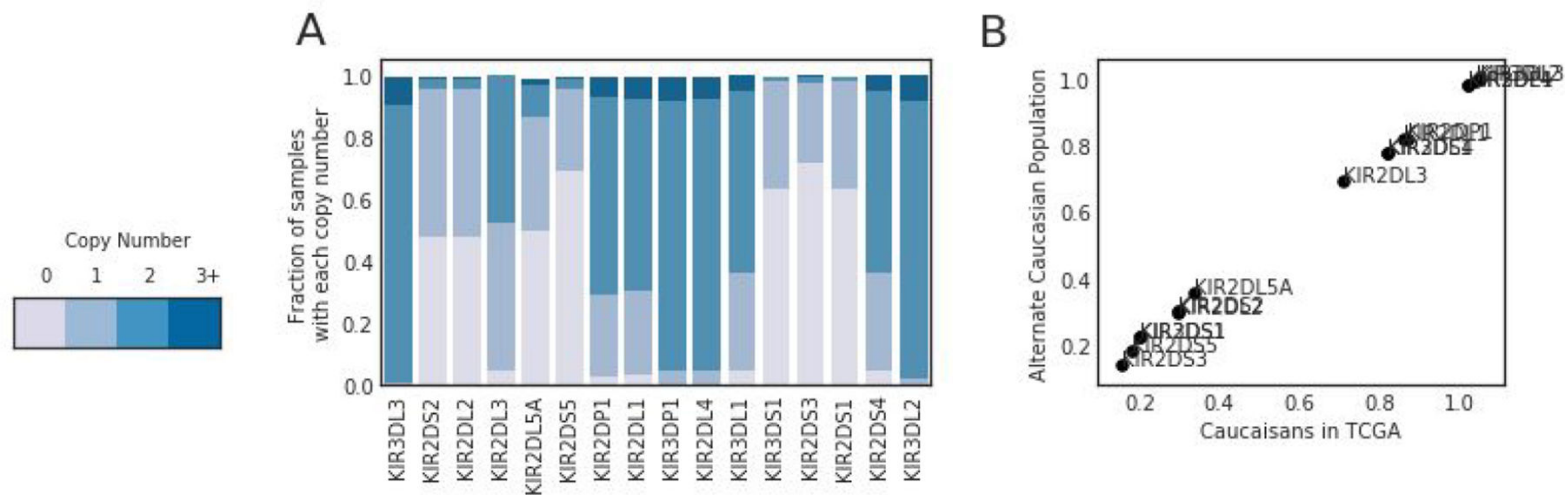
TCGA KIR copy number distribution and validation. (A) A stacked bar chart showing the fraction of patients with each copy number across all KIR genes. (B) A dot plot showing the comparison in gene frequency (average gene copy number per haplotype) within the European ancestry population of TCGA and an experimentally typed European ancestry population.

As anticipated, the distributions of copy number per KIR gene across the population are highly variable (Figure 6A). The anchor genes have two copies for nearly all individuals while non-anchor genes have a mixture of copy numbers. To validate our method computationally, we assessed correlation between known KIR copy number frequency against our algorithm. The results were very promising; there was a high correlation (R^2^ = .999) between ancestry-matched population frequencies of KIR haplotypes in TCGA and a recent study that used an experimental approach for typing^24^ (Figure 6B). This finding also suggests little or no germline KIR-based cancer predisposition; however, more comparisons with non-cancer populations will be required to make a definitive assertion.

### 3.5 KIR inhibitory gene burden correlates with survival in cervical and uterine cancer

**Figure 7.**
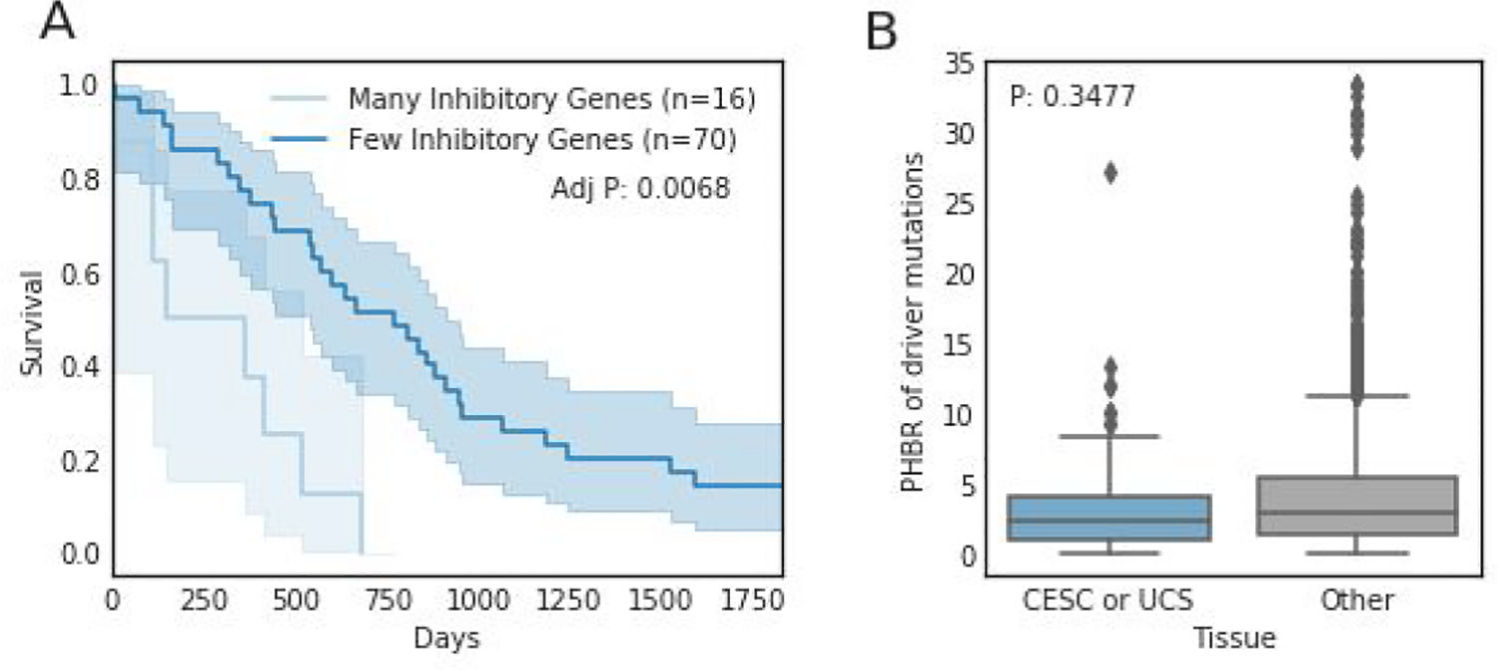
The impact of KIR copy number on tumor development phenotypes in CESC and UCS. (A) Kaplan-meier survival curves denoting the difference in survival between patients with more inhibitory genes than average and less inhibitory genes than average. (B) A boxplot showing the difference in MHC-I presentation of driver mutations between CESC and UCS.

KIR genes are divided into two functional categories: activating genes and inhibitory genes. Inhibitory genes bind to specific MHC-I ligands to inhibit the NK cell from attacking the MHC-I expressing cell^12,25^. Often in cancer, cells will down regulate their MHC-I molecules to avoid immune presentation of neoantigens. When this happens, there is no inhibition of the NK cells by the KIR, and NK cells attack. Activating genes have remained more elusive with their ligands and function mainly unknown^12^. They are believed to have evolved after the inhibitory genes and are non-essential to proper immune functioning. Since inhibitory genes are variable in copy number across individuals, we tested survival differences within tumor types for patients with high and low numbers of inhibitory gene copies. We found two tumor types, cervical squamous cell carcinoma (CESC) and uterine carcinosarcoma (UCS), with unadjusted p-values of less than 0.05 (P=0.000182 and P=0.0113, respectively). In both of these tumor types, patients with high numbers of inhibitory genes had lower survival rates, suggesting that NK cells were unable to defend against the tumor in these patients. Since these tumor types are physically co-localized and have similar immune infiltration profiles and survival rates (Figure S2), we analyzed these cohorts together to increase sample sizes (adj P=0.00612, Figure 7A).

To investigate why we found a significant survival difference in these two tumor types as compared to others, we explored the ability of their MHC-I to present observed driver mutations for recognition by the immune system^23^. Patients with CESC and UCS had better presentation of observed driver mutations to the immune system than other tumors (P=0.0034, Figure 7B), suggesting that the CESC and UCS tumors have immunosuppressive mechanisms at play. One possible mechanism for this immunosuppression is impaired antigen presentation, potentially via mutation^3^ or loss of heterozygosity in the HLA region^26^, allowing perpetuation of the tumor despite high affinity of observed drivers for the MHC-I. If MHC-I presentation on the cell surface is altered and T cells become less relevant, we expect that individuals with higher inhibitory KIR gene counts would have less ability to initiate an NK based attack against the tumor. These observations suggest that when NK cells are called to action, patients with higher NK cell inhibition may be less able to attack the cancer cells, resulting in a shorter survival time.

## 5. Conclusions

Though natural killer cells are increasingly being considered as targets for immunotherapy, little is understood about the role of KIR, their main receptor family, on tumor development. Here, we describe our effort to evaluate the copy number of KIR genes in a large cancer cohort to learn about their influence in relationship with MHC on tumor development. We demonstrate the value of algorithmically learning KIR copy number in a large population by uncovering a survival difference in CESC and USC based in the number of inhibitory genes carried by an individual. Due to batch effects in exome sequencing, the current method must be retrained on each new cohort of individuals. This limitation leaves us unable to validate many of our methods experimentally. Furthermore, our method does not provide allele calls and cannot be used to determine the copy number of small cohorts or individual patients. However, our analysis highlights the importance of KIR variability to tumor development and warrants further study of this complex locus.

## Supplementary Material

2
