# Supplementary material for: Computational KIR copy number discovery reveals interaction between inhibitory receptor burden and survival
Source: Pac Symp Biocomput. Author manuscript; Available in PMC 2019 Mar 14. (PMC6417817)
Supplement: 2 [file NIHMS999776-supplement-2.pdf]

Data S1: Code for this analysis can be found in the following repository: https://github.com/Rachelmarty20/KIR_development.

A **B**

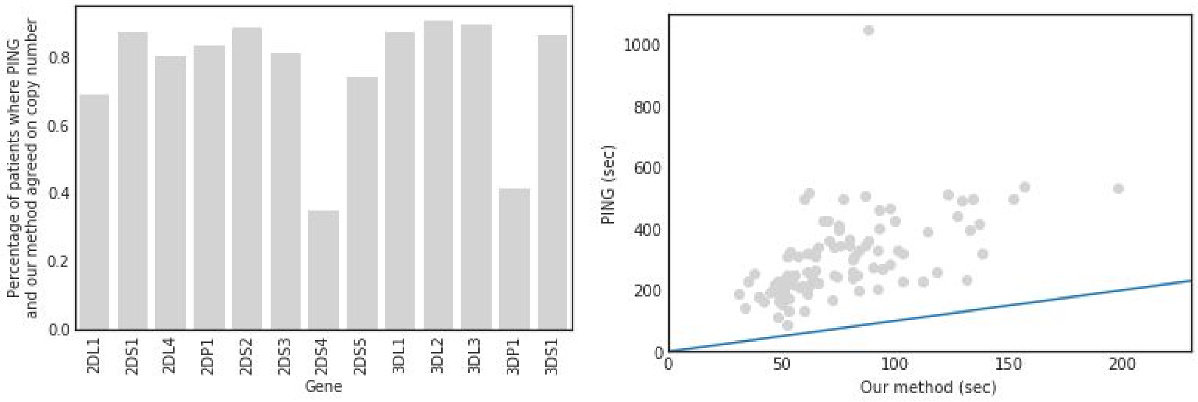

Figure S1: Comparison of our method with PING on a subset of TCGA patients. (A) A bar plot denoting the percentage of patients with exact copy number agreement for each gene. We found moderate agreement between the two tools. However, we cannot assess which tool is performing better without external validation. As a proxy, we compared the caucasian population frequencies with frequencies from PING and our method for the low agreement genes. For KIR2DS4, the caucasian population should have a frequency (number of gene copies per haplotype) of 0.78. While our method overestimated the frequency (0.87), PING severely underestimated the frequency (0.51). For KIR3DP1, the caucasian population should have a frequency of 0.98. Our method predicts a frequency of 1.02; however, PING again significantly overestimates the frequency (1.24). While these validations are imperfect, they suggest that our method is outperforming PING. (B) A scatter plot denoting the speed of both tools in seconds. The input to both of these methods was a bam file sliced from chromosome 19.

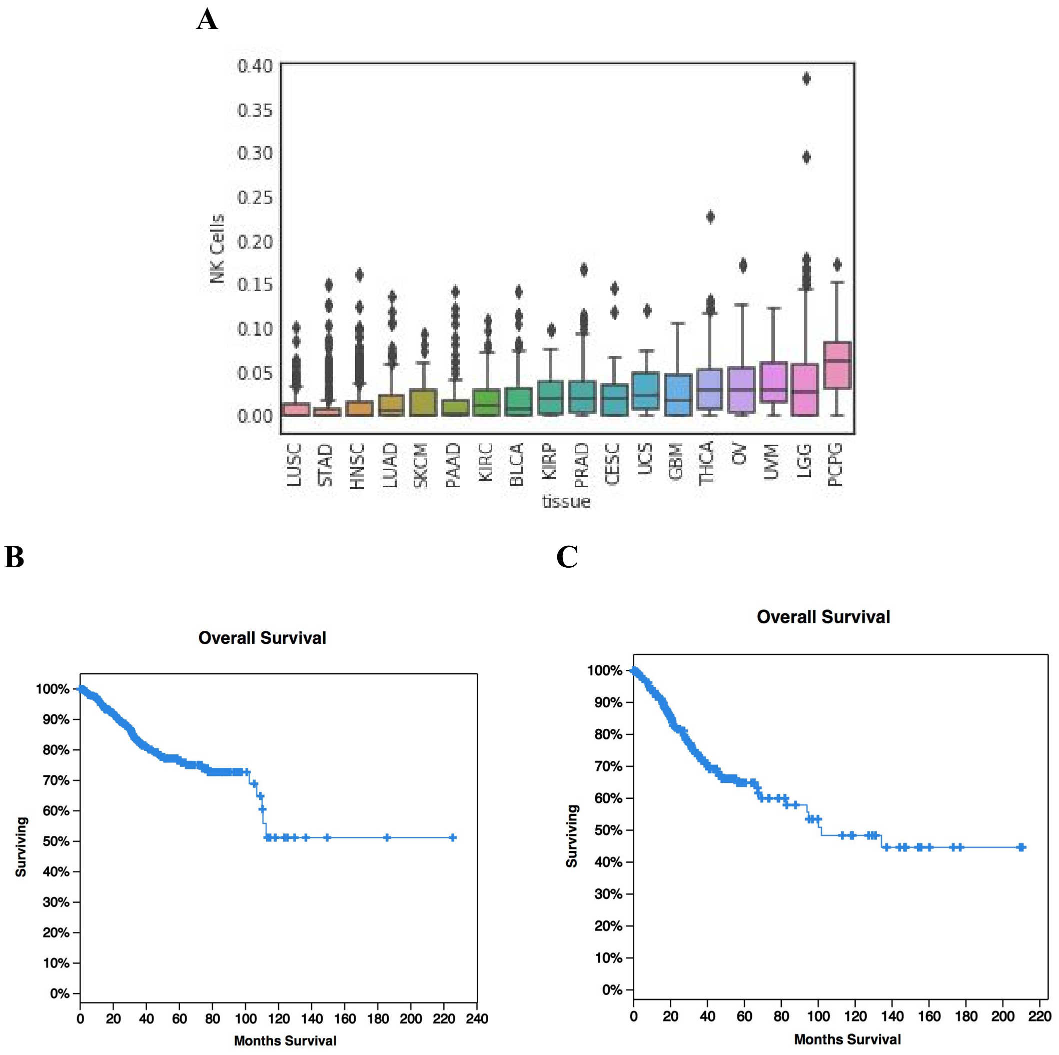

Figure S2. Similarity between CESC and UCS tumor types. (A) Box plots representing the distribution of natural killer (NK) cell infiltrate in each tumor type. The tissues are ordered by average infiltration, leaving CESC and UCS tightly clustered. (B-C) Survival curves for (B) UCS and (C) CESC.
